# Dopaminergic brainstem disconnection is common to pharmacological and pathological consciousness perturbation

**DOI:** 10.1073/pnas.2026289118

**Published:** 2021-07-23

**Authors:** Lennart R. B. Spindler, Andrea I. Luppi, Ram M. Adapa, Michael M. Craig, Peter Coppola, Alexander R. D. Peattie, Anne E. Manktelow, Paola Finoia, Barbara J. Sahakian, Guy B. Williams, Judith Allanson, John D. Pickard, David K. Menon, Emmanuel A. Stamatakis

**Affiliations:** ^a^University Division of Anaesthesia, Addenbrooke’s Hospital, University of Cambridge, Cambridge CB2 0SP, United Kingdom;; ^b^Department of Clinical Neurosciences, Addenbrooke’s Hospital, University of Cambridge, Cambridge CB2 0SP, United Kingdom;; ^c^Division of Neurosurgery, School of Clinical Medicine, Addenbrooke’s Hospital, University of Cambridge, Cambridge CB2 0SP, United Kingdom;; ^d^Department of Psychiatry, School of Clinical Medicine, University of Cambridge, Cambridge CB2 0SZ, United Kingdom;; ^e^Behavioural and Clinical Neuroscience Institute, University of Cambridge, Cambridge CB2 3EB, United Kingdom;; ^f^Wolfson Brain Imaging Centre, University of Cambridge, Cambridge CB2 0QQ, United Kingdom;; ^g^Department of Neurosciences, Addenbrooke’s Hospital, Cambridge University Hospitals National Health Service Foundation, Cambridge CB2 0SP, United Kingdom

**Keywords:** brainstem, consciousness, disorders of consciousness, dopamine, neurotransmitter

## Abstract

Clinical research into consciousness has long focused on cortical macroscopic networks and their disruption in pathological or pharmacological consciousness perturbation. Despite demonstrating diagnostic utility in disorders of consciousness (DoC) and monitoring anesthetic depth, these cortico-centric approaches have been unable to characterize which neurochemical systems may underpin consciousness alterations. Instead, preclinical experiments have long implicated the dopaminergic ventral tegmental area (VTA) in the brainstem. Despite dopaminergic agonist efficacy in DoC patients equally pointing to dopamine, the VTA has not been studied in human perturbed consciousness. To bridge this translational gap between preclinical subcortical and clinical cortico-centric perspectives, we assessed functional connectivity changes of a histologically characterized VTA using functional MRI recordings of pharmacologically (propofol sedation) and pathologically perturbed consciousness (DoC patients). Both cohorts demonstrated VTA disconnection from the precuneus and posterior cingulate (PCu/PCC), a main default mode network node widely implicated in consciousness. Strikingly, the stronger VTA–PCu/PCC connectivity was, the more the PCu/PCC functional connectome resembled its awake configuration, suggesting a possible neuromodulatory relationship. VTA-PCu/PCC connectivity increased toward healthy control levels only in DoC patients who behaviorally improved at follow-up assessment. To test whether VTA–PCu/PCC connectivity can be affected by a dopaminergic agonist, we demonstrated in a separate set of traumatic brain injury patients without DoC that methylphenidate significantly increased this connectivity. Together, our results characterize an in vivo dopaminergic connectivity deficit common to reversible and chronic consciousness perturbation. This noninvasive assessment of the dopaminergic system bridges preclinical and clinical work, associating dopaminergic VTA function with macroscopic network alterations, thereby elucidating a critical aspect of brainstem–cortical interplay for consciousness.

## Significance

Understanding the neural bases of consciousness is of basic scientific and clinical importance. Human neuroimaging has established that a network of interconnected brain regions known as the default mode network disintegrates in anesthesia and after brain damage that causes disorders of consciousness. However, the neurochemical underpinnings of this network change remain largely unknown. Motivated by preclinical animal work and clinical observations, we found that across pharmacological (sedation) and pathological (disorders of consciousness) consciousness perturbation, the dopaminergic source nucleus, the ventral tegmental area, disconnects from the main nodes of the default mode network. As the severity of this dopaminergic disconnection was associated with default mode network disintegration, we propose that dopaminergic modulation may be a central mechanism for consciousness maintenance.

Delineating the neural underpinnings of consciousness is of basic scientific and clinical importance, both to understand its reversible suppression during sedation/anesthesia (1) and to allow informed treatment choices for patients with chronic disorders of consciousness (DoC) (2). Striking parallels between these perturbed states of consciousness have been characterized using human neuroimaging, which converge in particular on the default mode network (DMN). This prominent large-scale network progressively loses functional connectivity integrity with increasing severity in DoC (3, 4) and also with increasing depth of anesthesia (5, 6). In both pharmacologically and pathologically perturbed consciousness, key DMN nodes in the posterior cingulate cortex (PCC) and precuneus (7, 8) show disrupted brain-wide functional connectivity associated with loss of consciousness (9). These macroscopic phenomena have demonstrated some diagnostic and prognostic value for DoC patients and form empirical bases for contemporary theories of consciousness (4, 10–13). However, these cortico-centric perspectives have remained inherently unable to address whether specific neurochemical systems may mediate perturbed consciousness and the associated macroscopic network changes. The delineation of these neurochemical drivers of consciousness is key to the identification of amenable therapeutic targets in DoC and for the understanding of anesthetic mechanisms—and as such for the formulation of an integrated clinical account of consciousness.

To this end, preclinical work has focused in on the brainstem neurotransmitter nuclei based on findings across a wide range of experimental animal work utilizing anesthetic drugs and lesion approaches. The transmitter systems studied in this context range from glutamate and glycine, over orexin and histamine, to acetylcholine and the biogenic amines (14). Among them, in particular the dopaminergic system, has emerged as a candidate neurochemical driver of consciousness due to its consistent implication in preclinical animal studies and clinical DoC contexts.

Preclinically, Palmiter and colleagues demonstrated that dopamine-deficient mice were behaviorally unconscious but that retroviral restoration of dopaminergic signaling reversed associated behavioral deficits (15). The source of the relevant dopaminergic signaling was subsequently identified to be the ventral tegmental area (VTA), the main dopaminergic brainstem nucleus. Both optogenetic (16) and pharmacological VTA activation (17) acutely promote wakefulness in rodents. Critically, electrical and optogenetic (18) stimulation of the VTA (but not substantia nigra) can even reverse the sedative effects of propofol (19)—whereas lesions to the VTA lengthen recovery times following propofol anesthesia in rodents (20). More broadly, methylphenidate-induced reversal of propofol anesthesia in rats is also suggested to act primarily via a dopamine-dependent mechanism (21, 22). These preclinical findings implicate dopaminergic signaling from the VTA as a tonic regulator of wakefulness.

While equivalent experiments cannot be carried out in humans due to their invasiveness, the relevance of dopamine has equally been revealed in clinical settings through reports of beneficial effects of various dopaminergic agonists in DoC patients. These drugs include levodopa (23), bromocriptine (24), methylphenidate (25), and amantadine (26, 27), with further promising results from past and ongoing small-scale trials of apomorphine (28). The corresponding and long-standing idea of dopaminergic dysfunction in DoC (29) is corroborated by a recent ^11^C-raclopride positron emission tomography (PET) study which indicated impaired presynaptic dopamine release in minimally conscious state (MCS) patients (30). However, despite this dopaminergic focus which parallels the preclinical implications of dopamine, research in humans has left unanswered whether pathological and pharmacological consciousness perturbations may arise due to impaired function of the main dopaminergic nucleus, the VTA. Consequently, the characterization of VTA function in humans with consciousness perturbations holds critical translational potential and could strongly enhance ongoing dopaminergic drug trials.

Explicitly, dysfunction of the VTA could also underpin macroscopic network alterations consistently observed in states of lowered consciousness, since dopamine acts as a neuromodulator (31–33). Neuromodulation is the process by which diffusely released nonclassical transmitters, such as dopamine, can alter intrinsic electrochemical and synaptic properties of neurons (32) and thereby change input–output relationships at all scales of brain organization (33, 34). This allows the finite number of anatomical connections in the brain to integrate into diverse meso- and macroscopic functional configurations (32, 33). As dopamine is diffusely released from the VTA to almost all cortical structures (31), dopaminergic neuromodulation is a strong candidate to mediate macroscopic network-level effects. This has been demonstrated for catecholaminergic manipulation more broadly in healthy individuals using functional MRI (fMRI) (35, 36) and is corroborated specifically for dopamine by the observation that posteromedial D2/D3 receptor occupancy measured with [^18^F]-fallypride PET is associated with whole-brain DMN integrity in healthy individuals (37). Critically, these dopaminergic effects have neither been tested in pharmacological and pathological consciousness perturbation despite the many implications of dopamine nor have they been associated with the dopaminergic source nucleus, the VTA. Conversely, rather than representing the loss of a crude “activating signal” (38), VTA dysfunction could reflect altered neuromodulatory environments, which may precipitate neural phenomena such as DMN disintegration in perturbed consciousness. As such, delineating relevant VTA function holds the potential to translationally scale insights from experimental models of VTA function to human in vivo neuroimaging contexts of altered networks.

We consequently aimed to explore how this dopaminergic nucleus behaves in perturbed consciousness in resting-state fMRI (rs-fMRI) data from healthy volunteers undergoing propofol sedation (*n* = 24) and patients with chronic DoC (*n* = 22) using an anatomically and histologically validated VTA region of interest (39). We chose rs-fMRI because of its better spatial resolution in comparison to PET and as the latest guidelines by UK and US clinical bodies recommend the use of this noninvasive imaging technique in DoC patient management (40). Explicitly, we hypothesized that 1) alterations in VTA functional connectivity would occur across both reversible and pathological perturbation of consciousness, that 2) these alterations should relate to whole-brain connectivity changes, behavior, and/or outcome, and that 3) VTA functional connectivity may be affected by a dopaminergic agonist.

## Results

### The VTA Loses Precuneus and Posterior Cingulate Connectivity during Propofol Sedation and in DoC.

**Fig. 1.**
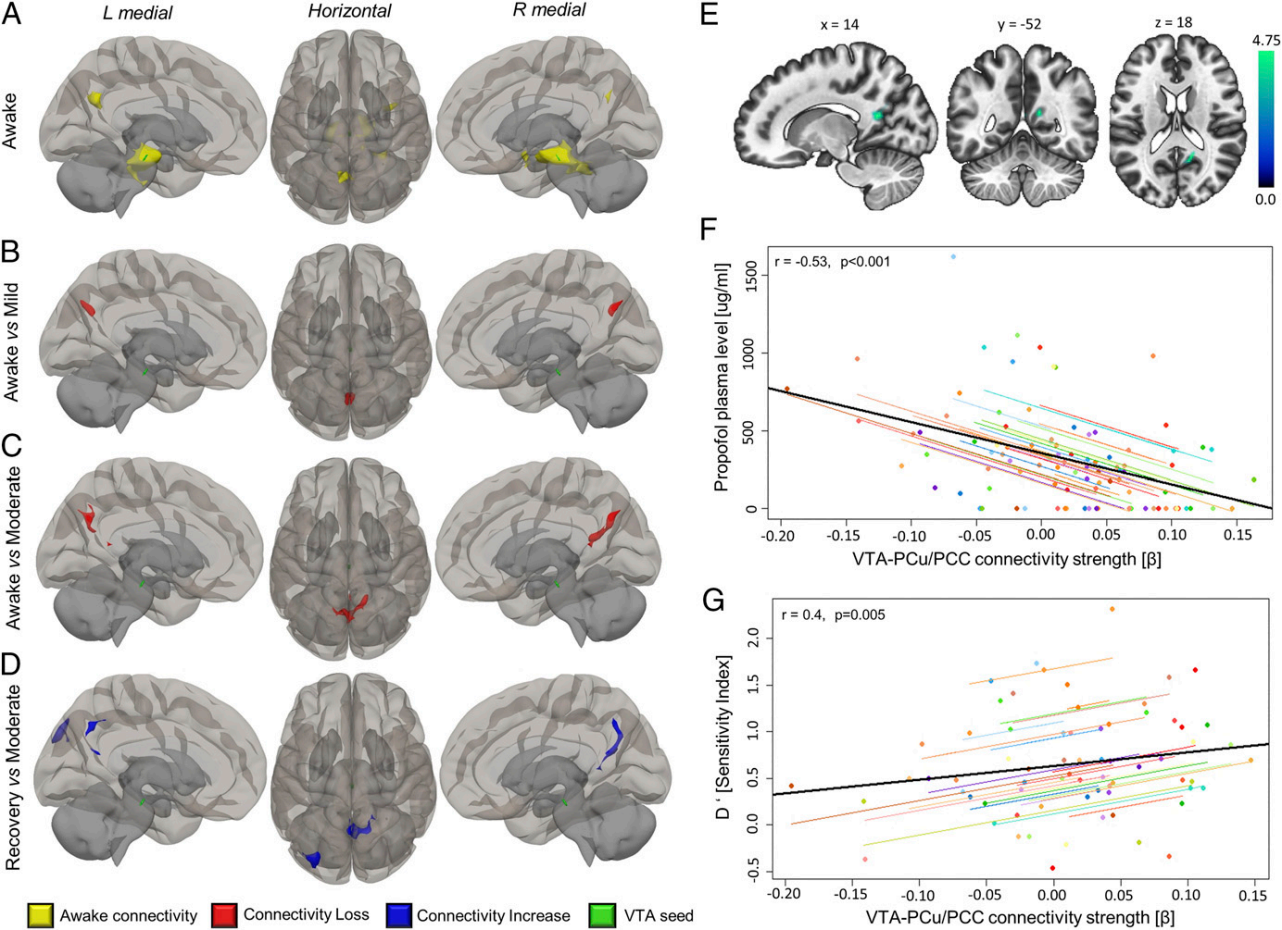
VTA disconnects stepwise and reversibly from PCu/PCC in propofol sedation. (*A*) In awake participants, the VTA ROI showed resting-state functional connectivity to PCu/PCC as well as hippocampal, insular, and cerebellar areas (Table 1). Under mild (*B*) and moderate (*C*) propofol, the VTA showed a stepwise loss of connectivity specifically with precuneus and posterior cingulate. (*D*) In recovery, connectivity to precuneus and PCC was regained (blue). (*E*) Anatomical slice display, centered on peak MNI coordinates, of cluster from *C*, which was used for extraction of subject-specific VTA-to-PCu/PCC connectivity values. (*F*) The higher the effective propofol dosage was in participants’ plasma, the more disconnected the VTA was from the PCu/PCC cluster (i.e., lower connectivity) across all experimental conditions. (*G*) The strength of this connectivity was positively predictive of how reliably participants were able to discriminate novel and familiar stimuli from a semantic processing task performed at each sedation level, measured as the sensitivity index d′. The colored dots in graphs are individual participants. The black line is the overall regression line without participant variable. Statistical thresholds for connectivity changes were voxel level *P* < 0.005 (uncorrected) and cluster level *P* < 0.05 (FWE corrected). Brains are in neurological orientation, that is, “L” is left. Renderings were made using the CONN toolbox three-dimensional template.

As the VTA’s involvement in sedative mechanisms has been established in preclinical models (41), we began by assessing the VTA’s functional connectomic changes during propofol sedation using the histologically characterized VTA region of interest (ROI) from the Harvard ascending arousal network atlas [HAAN (39)]. In awake volunteers, the VTA showed functional connectivity to the precuneus, PCC, brainstem, cerebellum, insula, and hippocampus (Fig. 1*A* and Table 1), consistent with previous reports by Bär and colleagues (42). However, upon propofol administration, whole-brain contrasts between awake versus mild (Fig. 1*B*) and awake versus moderate sedation (Fig. 1*C*) showed that the VTA lost connectivity exclusively with a cluster in the precuneus and PCC. The connectivity of the VTA to these areas re-emerged during recovery from sedation (Fig. 1*D*). The connectivity strength of the VTA to the precuneus and PCC cluster was negatively correlated with participants’ plasma propofol concentrations across all experimental conditions (r = −0.53, *P* < 0.001; Fig. 1 *E* and *F*).

**Table 1.** VTA awake connectivity and connectivity changes in propofol sedation and DoC compared to respective control conditions

Condition/ contrast	Δ connectivity change	Anatomical regions (CONN atlas)	Peak MNI coordinates	Cluster size	Cluster p (FWE corrected)
Awake volunteers	—	Brainstem, Cereb45 l+r, Cereb6 r, Hippocampus l+r, pPaHC l+r, pTFusC r, TOFusC r, aPaHC l+r, Amygdala r	+00 –24 −18	1,975	0.000
		**Precuneus, PC**	−04 –64 +26	192	0.000
		IC r, Amygdala r	+32 +00 –20	111	0.003
Awake > moderate (RL3)	↓Loss	**Precuneus, PC**	+14 –52 +18	424	0.000
Awake > mild (RL2)	↓Loss	**Precuneus**	+00 –68 +32	169	0.035
Recovery > moderate (RL3)	↑Gain	**Precuneus, PC** sLOC l, OP l	+00 –56 +20	317	0.000
			−36 −84 +38	213	0.009
Healthy controls	—	Brainstem, Thalamus l+r, pPaHC l+r, Cereb3 l+r, Cereb45 l	−02 –22 −18	1,226	0.000
		**Precuneus**, **PC**	+04 –46 +18	268	0.000
Awake > DoC	↓Loss	**FP** l, PaCig l	−22 +42 +22	235	0.036
		**Precuneus, PC**	+10 –46 +24	223	0.046
DoC > awake	↑Gain	LG l+r, OFusG l, Cereb1,45,6,8,9 l+r, TOFusC l+r, Ver45,6,7,10, OFusG r, pPaHC	−34 –80 −12	5,204	0.000
		Hippocampus r, TP r, Amygdala r, PP r, aSTG r, pMTG r, IC r, aMTG r	+52 –02 −18	1,149	0.000
		Hippocampus l, Brainstem	−14 –18 −18	523	0.000

CONN atlas labels, peak MNI coordinates, cluster extent, and FWE-corrected *P* values are reported. DMN regions are in bold. Awake connectivity was thresholded at *P* < 0.001 voxel level (uncorrected) and contrasts at *P* < 0.005 (uncorrected) with *P* < 0.05 cluster level (FWE corrected). ↓Loss corresponds to decreased functional connectivity and ↑Gain to increased connectivity in comparison to control group.

**Fig. 2.**
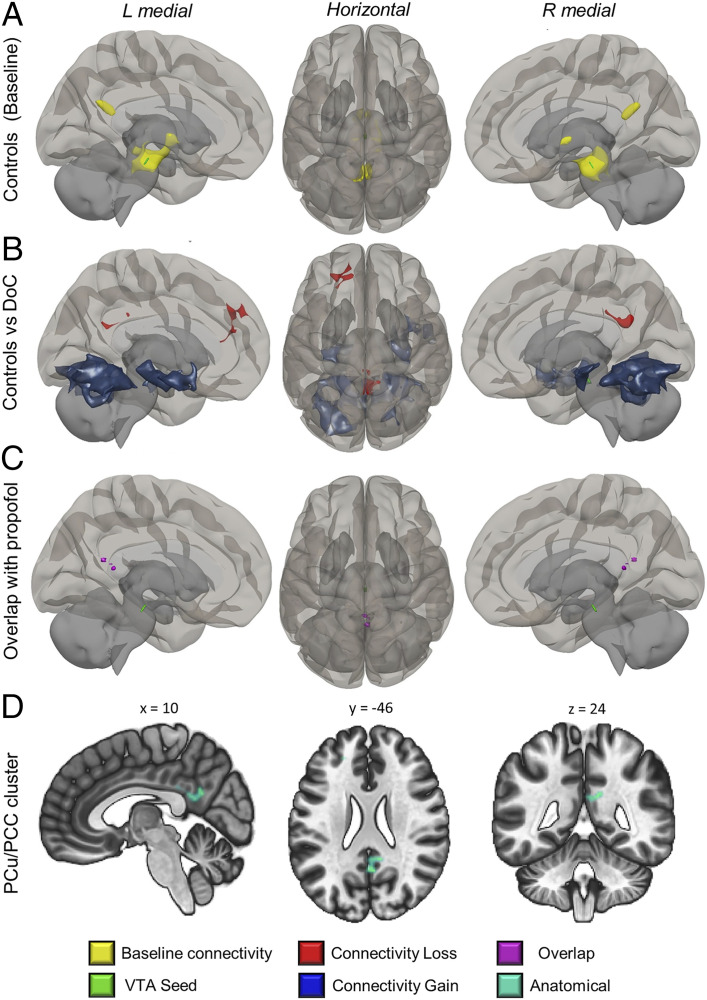
Loss of VTA connectivity to precuneus and posterior cingulate in DoC. (*A*) In the control cohort used for comparisons with the DoC patients, the VTA also showed precuneus and posterior cingulate connectivity with an additional cluster in the thalamus. (*B*) In a contrast of patients to these awake controls, VTA connectivity losses were observed with PCu/PCC and mesiocortical regions, with concomitant subcortical gains (Table 1). (*C*) At a lowered voxel threshold, the posterior disconnection clusters from sedation and DoC datasets spatially overlapped. (*D*) Display of posterior region from *B* on anatomical slices centered on peak MNI coordinates. This cluster in *D* was used for connectivity strength extraction. Images are in neurological orientation, that is, “L” is left.

The healthy control group used for comparisons with the DoC patients showed equivalent awake connectivity of the VTA but displayed a small additional connectivity cluster in the thalamus (Fig. 2*A*). When DoC patients were compared to controls, the VTA showed reductions in functional connectivity with the precuneus and PCC, resembling the changes seen in propofol sedation (Figs. 1 *A* and *B* and 2*B*). In addition, we also found reductions in connectivity of the VTA to the mediofrontal cortex (another hub of the DMN). The VTA in DoC patients also showed some increases in functional connectivity to other brain areas, largely to subcortical regions and the hippocampus (Fig. 2*B* and Table 1). The clusters of VTA disconnection that we observed in both propofol sedation and DoC cohorts overlapped spatially in the precuneus and posterior cingulate (clusters hereafter: PCu/PCC) across both conditions.

### VTA–PCu/PCC Connectivity Is Associated with Relevant Behavior in Propofol Sedation and in DoC Patients.

To answer whether the VTA’s connectivity loss to PCu/PCC was associated with behavior, we extracted β-coefficients from the general linear model for VTA–PCu/PCC connectivity and correlated these with relevant behavioral measures for each cohort.

For the propofol cohort, we tested whether stimuli presented for a semantic decision task during the awake and sedated states were subsequently identified as familiar or novel after recovery from sedation (see *Materials and Methods*). The participants’ ability to correctly classify stimuli as novel or familiar was quantified as the sensitivity index d′ (d prime), a proxy for explicit memory formation with conscious cognitive access (43). We found that the d′ score covaried positively with VTA–PCu/PCC connectivity during propofol-based perturbation of consciousness (*r* = 0.4, *P* < 0.001, Fig. 1*G*).

Similar behavioral measurements in patients with DoC are impossible. We nevertheless aimed to assess whether VTA–PCu/PCC connectivity may be related to behavior or outcome.

There was no significant correlation with the coma recovery scale—revised (CRS-R; *r* = 0.2, *P* = 0.373) or its arousal subscore (*r* = 0.41, *P* = 0.066) at time of scan. However, we more closely examined the patients within our sample who had follow-up scans (*n* = 7, mean elapsed time 509 ± 131 d). Among this subsample, two patients improved in terms of CRS-R and remain alive, whereas the remaining five further deteriorated and subsequently deceased. Although the small subsample size makes meaningful statistics impossible, it is relevant to report that the two alive and improved patients showed a re-emergence of positive VTA–PCu/PCC connectivity at their second imaging assessment, improving from negative VTA–PCu/PCC connectivity recorded at the previous assessment. In contrast, the patients who deteriorated (lower CRS-R scores) and deceased after the imaging follow-up had maintained and/or worsened negative VTA–PCu/PCC connectivity (*SI Appendix*, Table S1).

### VTA–PCuu/PCC Connectivity Strength Is Associated with PCu/PCC Whole-Brain Connectome Disintegration.

The areas of disconnection we observed for the VTA were in the PCu/PCC and displayed resting connectivity to key nodes of the DMN in whole-brain analyses in both control cohorts (*SI Appendix*, Fig. S1 and Table S2). As both DMN intrinsic connectivity and its functional relationships with other brain regions are commonly altered during perturbations of consciousness, we next asked whether changes in this large-scale network could be driven by altered VTA–PCu/PCC connectivity—possibly reflecting altered neuromodulation of the PCu/PCC, precluding correct functional circuit assembly (32). Because neuromodulation itself cannot be measured with any single-modality neuroimaging technique, we sought to approximate a VTA neuromodulatory relationship by different means. We examined whether VTA–PCu/PCC connectivity strength in perturbed consciousness covaried with brain-wide connectivity alterations between the PCu/PCC and other brain regions (within and outside the DMN) in perturbed consciousness (see *Materials and Methods* and *SI Appendix*, Fig. S2 for full “downstream” brain-wide connectivity changes).

**Fig. 3.**
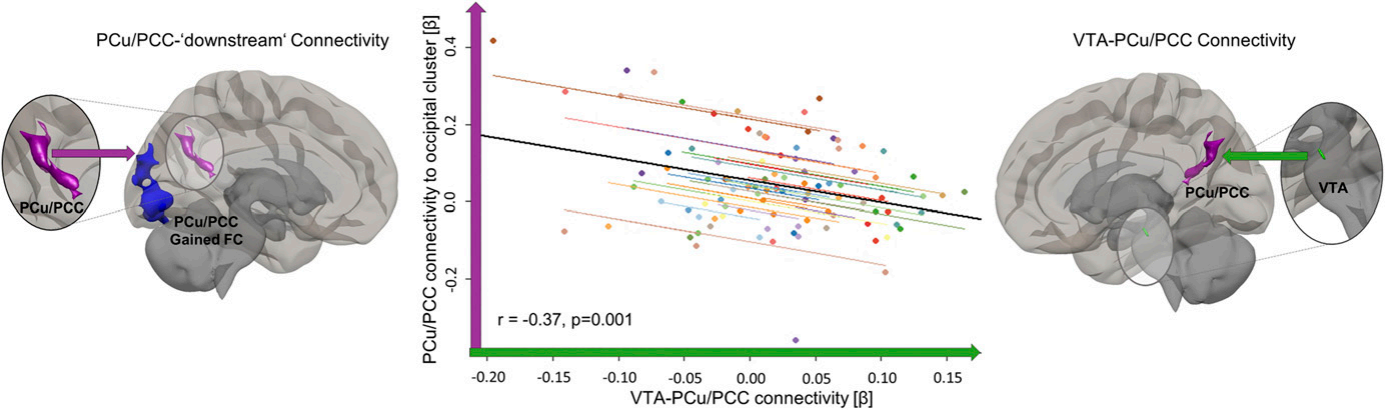
VTA–PCu/PCC connectivity strength is associated with its PCu/PCC target’s whole-brain connectivity alteration in propofol sedation. Across all conditions in the propofol experiments, repeated measures correlations revealed that the stronger the connectivity (green arrow/axis) between the VTA (green ROI, magnified) and its PCu/PCC target (magenta ROI, magnified), the weaker the connection (magenta arrow/axis) between this PCu/PCC target and downstream beyond-DMN occipital gains (blue cluster) was. Downstream connectivity gains were characterized by using the PCu/PCC cluster originally identified in population-level contrasts of VTA connectivity (Fig. 1 *C* and *E*) in new seed-to-voxel analyses (reference *SI Appendix*, Fig. S2*B* for “downstream” seed-to-voxel analyses). The dot color represents the individual participant. The black line is overall regression line, ignoring the participant variable. All masks used for connectivity extraction were thresholded at voxel level *P* < 0.005 (uncorrected) and at cluster level *P* < 0.05 (FWE corrected). FC = functional connectivity.

In the case of propofol sedation, the PCu/PCC cluster from which the VTA disconnected showed no reductions in connectivity with any part of the brain that reached significance. However, it showed significant increases in connectivity to areas that are not part of the canonical DMN (Fig. 3 and *SI Appendix*, Fig. S2*B* and Table S3). In repeated measures correlations across all sedation conditions, PCu/PCC connectivity to these predominantly visual non-DMN areas was negatively correlated with VTA–PCu/PCC connectivity strength (r = −0.37, *P* = 0.001) (Fig. 3).

**Fig. 4.**
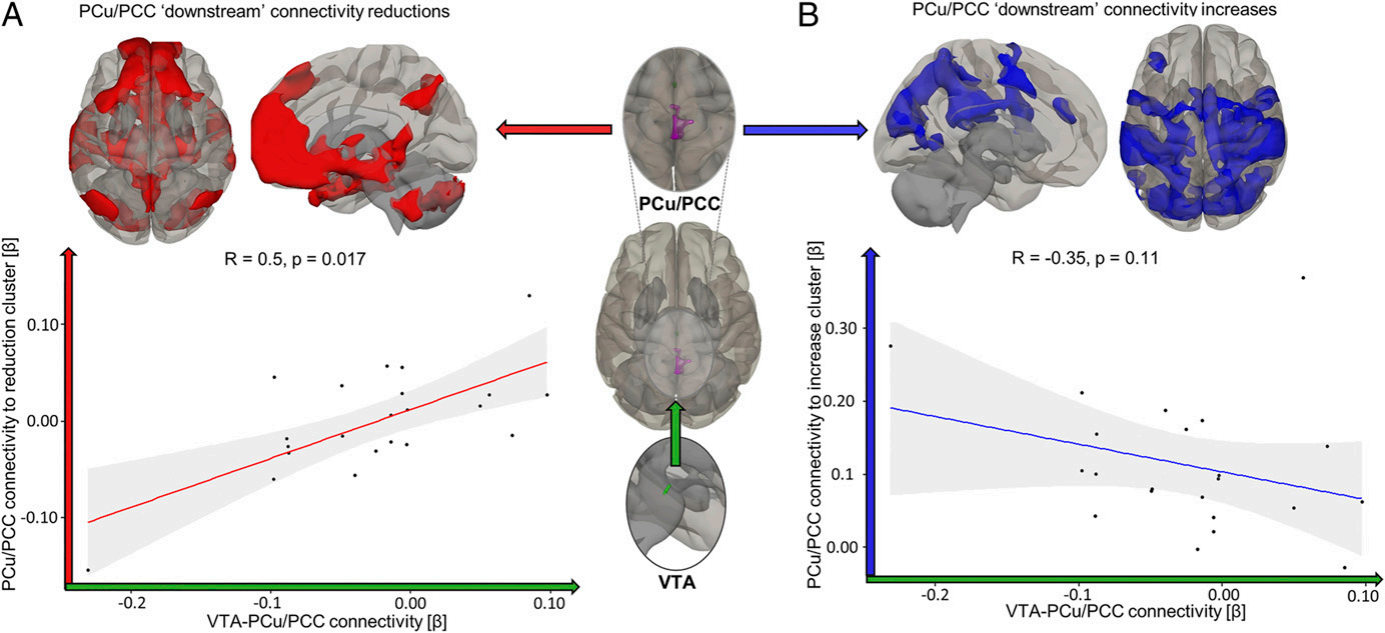
VTA–PCu/PCC connectivity is associated with the strength of PCu/PCC connectivity to DMN areas in DoC patients. (*A*) The VTA’s connectivity strength to its PCu/PCC target (green arrow/axis) was positively associated with the PCu/PCC’s own connectivity strength to “downstream” DMN-centric areas with which it lost connectivity at population level (red arrow/axis). Expressly, the more VTA–PCu/PCC connectivity was preserved for each patient, the more its PCu/PCC connectivity strength maintained a more awake-like DMN appearance. (*B*) The negative association of VTA–PCu/PCC connectivity strength with PCu/PCC connectivity to a cluster of increased connectivity (such as observed for propofol sedation) did not reach significance (blue arrow/axis). Correlations of connectivity strengths between respective seed and target masks used Spearman’s rank. Reference *SI Appendix*, Fig. S2*A* for “downstream” seed-to-voxel analyses from which masks used for β-value extraction were extracted at voxel level *P* < 0.005 (uncorrected) and at cluster level *P* < 0.05 (FWE corrected) thresholds. 95% CIs in gray.

The PCu/PCC cluster in patients with DoC showed wide-ranging reductions in connectivity, predominantly with areas classically identified as DMN regions (Fig. 4*A*, red and *SI Appendix*, Fig. S2*A* and Table S3). Concomitantly, the PCu/PCC also showed gains in connectivity to occipital regions in the DoC patients (similar to changes seen with propofol sedation in healthy volunteers), with additional connectivity gains to sensorimotor, precentral, postcentral, and anterior cingulate cortices (Fig. 4*B*, blue). The strength of PCu/PCC connectivity to clusters with which it lost connectivity covaried directly with changes in VTA–PCu/PCC connectivity (*r* = 0.5, *P* = 0.017; red, Fig. 4*A*), indicating a potential modulatory role of VTA–PCu/PCC connectivity for intra-DMN connectivity. The association of VTA–PCu/PCC connectivity with PCu/PCC connectivity to areas beyond the classical DMN did not reach significance in DoC patients (r = −0.35, *P* = 0.11; Fig. 4*B*, blue).

### Methylphenidate Increases VTA–PCu/PCC Connectivity in a Sample of Traumatic Brain Injury Patients Without DoC.

Finally, we hypothesized that if VTA–PCu/PCC connectivity is indeed a correlate of dopaminergic neuromodulation, it should be altered by a dopaminergic agonist (27). We tested this hypothesis in a separate cohort of traumatic brain injury/diffuse axonal injury patients (*n* = 12) who did not have DOCs but participated in rs-fMRI data collection in two separate sessions 2 to 4 wk apart: once with a placebo and once with administration of 30 mg methylphenidate, a dopaminergic and noradrenergic agonist (see *Materials and Methods*). VTA connectivity to the cluster of PCu/PCC disconnection observed in DoC patients (Fig. 2) revealed that, as hypothesized, VTA–PCu/PCC connectivity was significantly higher (*t*(11) = −1.957, *P* = 0.038) in the methylphenidate condition (M = 0.065 ± 0.018) compared to placebo administration (M = 0.011 ± 0.020) (*SI Appendix*, Fig. S3).

## Discussion

When compared to healthy awake conditions, both pathological and pharmacological states of lowered consciousness demonstrate a substantial disruption of VTA connectivity to the PCu/PCC, a central node of the brain’s DMN. This in vivo evidence of a consciousness-relevant dopaminergic system dysfunction common to DoC patients and sedation is critically also associated with a loss of specificity in the PCu/PCC’s whole-brain functional connectivity—the stronger VTA–PCu/PCC connectivity remained, the less the PCu/PCC’s normal connectivity pattern was perturbed. This indicates that disrupted dopaminergic neuromodulation of this main DMN node may at least partially underpin the commonly observed DMN disruptions in human perturbed states of consciousness (2, 44). We further demonstrate that this VTA–PCu/PCC connectivity strength can be modulated by the dopaminergic agonist methylphenidate.

We observed a stepwise and reversible VTA–PCu/PCC disconnection during propofol sedation, which is in keeping with a similar, but undiscussed, finding during dexmedetomidine [α-2 adrenergic agonist administration (45)]. However, our data link this change in connectivity to propofol plasma levels and behavioral measures of cognitive access. Above all, however, we demonstrate that this VTA disconnection from the PCu/PCC is equally found in chronic DoC patients. This provides a neuroimaging demonstration of a dopamine-specific parallel between pathological and pharmacological perturbation of consciousness.

In signaling terms, the PCu/PCC’s richness in D1 (46) and D2/D3 dopamine receptors (47) allows its ready modulation by dopamine, suggesting that the VTA functional disconnection may have physiological consequences: as preclinical models of chronic traumatic brain injury (TBI) (48) and of anesthetic treatment (49) variously show reduced dopamine levels, lowered VTA–PCu/PCC connectivity in rs-fMRI may coincide with or represent lowered dopaminergic tone and therefore altered modulation of targets. The fact that dopamine levels in rodents only elevate back to baseline in recovery from sedation (49) supports the assertion that the observed re-emergence of VTA–PCu/PCC connectivity in recovery from sedation could indeed indicate the restabilization of dopaminergic tone. This would be consistent with sudden increases in VTA connectivity having recently been suggested to have a mechanistic influence in “active” emergence from propofol anesthesia in humans (50). Despite the small sample size, the observed re-emergence of positive VTA–PCu/PCC connectivity only in those who improved and remain alive in our sample of DoC patients (in contrast to those who did not improve and subsequently deceased) suggests a possibly similar mechanism in DoC. Indeed, the recently demonstrated deficit of presynaptic dopamine release suggests that dopaminergic agents which affect behavioral improvements (51) may stabilize VTA functionality and connectivity and/or compensate for the neuromodulatory disruption we approximated. The authors of the related study equally hypothesized the deficit revealed with [^11^C]-raclopride-PET to arise from the brainstem, presumably the VTA (30). As their analysis, however, restricted itself to dopamine in the striatum and thalamus, they provided no data on the PCu/PCC. However, based on the VTA’s global projections, its release deficit should likewise affect cortical regions such as the PCu/PCC. As [^11^C]-raclopride binding is dominated by a high density of dopamine receptors in the striatum (47), we suggest that fallypride and SCH23390 PET with concurrent rs-fMRI would provide a more suitable tool to explore whether alterations in cortical dopamine receptor binding in DoC patients are associated with our observed connectivity deficit (52). Preclinical work by Taylor et al. and Kenny et al. (18, 22, 53) has provided strong evidence of a link between the arousal effects of the VTA and the D1 receptor—whereas some recent work has instead linked VTA dopamine arousal effects to the D2 receptor (17). Further preclinical studies and work in humans should therefore aim to tease apart whether these receptors have divergent or convergent functions in this context. To this end, ^18^F-Fallypride PET with concurrent rs-fMRI carried out by Nagano-Saito et al. has revealed that dopaminergic D2/D3-mediated modulation of the PCu/PCC is functionally important, as D2/D3 receptor binding in posterior cortical regions (coincident with the PCu/PCC) is highly correlated to DMN intranetwork connectivity strength in healthy adults (37).

Our association of VTA–PCu/PCC connectivity with the PCu/PCC cluster’s whole-brain connectivity changes noninvasively replicates and extends these findings by Nagano-Saito and colleagues. In covariance with VTA–PCu/PCC connectivity strength, the PCu/PCC connectome becomes less specialized, evidenced by the fact that increases in connectivity occur with commonly anticorrelated and losses to normally correlated regions (compare *SI Appendix*, Fig. S4). Together, these observations suggest that the neurobiological mechanisms by which both pharmacological intervention and DoC pathology alter cortical brain-wide connectivity may be dopamine centric. Our findings suggest that VTA–PCu/PCC connectivity may sustain the typical architecture of the DMN, putatively affecting how the functional connectivity profile of normal, waking consciousness can be orchestrated on the PCu/PCC structural connectome. If this VTA-mediated functional diaschisis is further substantiated with simultaneous PET and rs-fMRI, our seed-based rs-fMRI approach could become a noninvasive and clinically useful in vivo tool to identify consciousness-specific dopaminergic modulatory deficits—particularly as rs-fMRI has been suggested by both UK and US clinical bodies to be incorporated into the clinical management of DoC patients (40). This approach could feasibly be extended to other transmitter systems and disorders. Given the up-regulation of the VTA–PCu/PCC connectivity during methylphenidate administration, the monitoring of this connectivity may further prove a valuable pharmacodynamic fMRI marker for ongoing drug trials. We, however, acknowledge that as the TBI patients who received methylphenidate did not have DoC, we may be observing an up-regulation of functionally dormant but structurally intact VTA connectivity.

For clinical practice and research, this parallel between pharmacological and pathological consciousness disruption centered on the dopaminergic brainstem incentivizes a conceptual refinement to current theories of DoC. Elaborating on the idea of coma as a brainstem structural disconnection syndrome (54), the more heterogenous strata of DoC may be best understood on a spectrum of structural and functional brainstem disconnections, which may have diaschisis-like consequences on particularly important cortical network nodes. Critically, functional deficits may, however, be reversible as observed in recovery from propofol and in the two improved DoC patients whose VTA–PCu/PCC connectivity re-emerged—and may be amenable to therapeutic intervention as evidenced by this connectivity’s increase during methylphenidate administration. Dormant but structurally intact brainstem neuromodulatory connections could be a key therapeutic target for dopaminergic and other agonists. In future work, it will be imperative to tease apart whether the VTA is only functionally abeyant but structurally intact in DoC patients, as this would clarify which treatment strategies and which targets are amenable (i.e., postsynaptic versus presynaptic mechanisms). We suggest that despite the striking parallel between propofol sedation and DoC centered on the VTA, this mapping of structure–function brainstem deficits should be extended to include brainstem nuclei of other transmitter phenotypes, given the sporadic successes of nondopaminergic therapies (51) in DoC. Expressly, individual patients may have equally individual brainstem nucleic connectivity (and thus transmitter system) deficits. Even in our sample of DoC patients, brain-wide PCu/PCC connectivity alterations also included sites of nondopaminergic brainstem nuclei of glutamatergic, cholinergic, and serotonergic transmitter phenotypes. Patients’ neuromodulatory deficits may therefore overall be more complex and could vary relevantly between patients both in terms of source nuclei and cortical and subcortical targets. Distinguishing how different portions of the brainstem nuclei interact with posterior elements of the DMN as we show, but also with components of the anterior forebrain mesocircuit, could redefine how their “arousal signals” produce the synthesis of the preconditions of consciousness (29). This would redefine the previous macroscopic reports of whole brainstem disconnection from cortical networks (55) into a potential clinical tool to identify which transmitter systems show deficits for a specific patient, providing a rational basis for precision medical therapeutic interventions in DoC.

Despite the potential involvement of other nuclei in DoC more broadly, the consistency of VTA disconnection across pharmacological and pathological perturbation of consciousness in our samples—together with the wide-ranging preexisting implications of dopamine—suggest that dopaminergic neuromodulation from the VTA may be a particularly important component of consciousness maintenance. Including in vivo biomarkers of the integrity of VTA and dopaminergic system function such as the connectivity demonstrated here could significantly enhance the ability of ongoing drug trials and existent theories of DoC to account for dopamine’s efficacy in improving neural and behavioral symptoms of consciousness perturbation.

We acknowledge various limitations of our work. Firstly, our experiments lack the temporal resolution to delineate the temporal sequence of whether effects operate primarily at the VTA’s level rather than first occurring at the cortical level. We cannot exclude that the pharmacological and pathological effects in propofol and DoC may originate at the level of the PCu/PCC as previously suggested for perturbed consciousness (44)—and may only thereafter affect the VTA. However, as this cortical region is thought not to project to brainstem nuclei, effects at the level of the VTA may still precede and could still strongly influence those at the cortical level. Furthermore, as the VTA’s whole structural connectome in humans remains unclarified, it is unclear whether it structurally projects to the PCu/PCC per se (56)—although the diffuse nature of neuromodulation and the existence of dopamine receptors in the PCu/PCC suggest so. Additionally, the relatively short duration of scans and the lower spatial resolution of 3T MRI imaging limits the impact of our work (57). However, we accounted for motion, cardiac, respiratory, and physiological noise artifacts in our denoising procedure and have begun work to replicate these findings at 7T. Furthermore, the dysregulation of functional connectivity cannot yet conclusively be associated with a change in transmitter tone, despite the supporting findings from PET (37), related preclinical observations (19), and our findings with methylphenidate suggesting so. Further work will need to use multimodal fMRI and PET techniques with various dopamine receptor– and transporter-specific ligands to close this knowledge gap.

In conclusion, we provide evidence of a functionally relevant impairment in the dopaminergic system which is common to both pharmacologically and pathologically perturbed consciousness. This provides a possible translational bridge between preclinical and clinical observations concerning dopamine’s relevance for consciousness, as this connectivity’s disruption can account for alterations in macroscopic connectivity. The integrity of VTA–PCu/PCC connectivity could be a noninvasive in vivo biomarker of the dopaminergic modulatory conditions for consciousness. Its assessment could have prognostic and diagnostic uses in intensive care settings (and possibly beyond), with a particular potential to track pharmacological intervention effects. Altogether, these findings provide a stepping-stone toward understanding how neurochemical influences from the brainstem affect the rest of the brain to bring about consciousness—and incentivize more concerted neuroimaging explorations in this direction. Our work indicates that insights into these transmitter systems may hold particular promise for accelerating the translation of insights and treatments from benchside to bedside.

## Materials and Methods

### Participants and Data Acquisition.

A total of 20 healthy controls and 23 adults who were diagnosed as in unresponsive wakefulness syndrome (UWS) (*n* = 9) or MCS (*n* = 14) in line with current clinical guidelines were scanned using a Magnetom 3T Tim Trio (Siemens Healthcare) at the Wolfson Brain Imaging Centre, Addenbrooke’s Hospital, Cambridge. Structural T1-weighted acquisitions were made using a fast magnetization-prepared rapid gradient-echo (MP-RAGE) sequence (Repetition time (RT)= 2,300 ms, Echo time (TE) = 2.47 ms, 150 volumes at 1 × 1 × 1 mm^2^ resolution). Functional resting-state scans used an echo-planar interleaved descending sequence consisting of 32 slices (TR = 2,000 ms, Inversion time (TI) = 900 ms, TE = 30 ms, flip angle = 78°, 3 × 3 × 3.75 mm^2^ resolution) with a scan duration of 10 min. These 23 DoC patients (Table 2) were selected from a larger overarching dataset (*n* = 71). These were all recruited from specialized long-term care centers. Patients required a DoC diagnosis, written informed consent of participation from their legal representative/surrogate decision maker, and capability of being transported to Addenbrooke’s Hospital (Cambridge, United Kingdom) to be invited to this study. Exclusion criteria included any medical condition that made participation unsafe (decision made by clinical personnel blinded to study aims) or any unsuitability for the MRI scanner environment (e.g., non-MRI safe implants), significant preexisting mental health problems, or insufficient English premorbid language ability. Patients spent a total of 5 d (including arrival and departure days) at Addenbrooke’s Hospital. After admission, each patient underwent clinical and neuroimaging testing. Patients were not sedated at time of scan. CRS-R assessments were recorded at least once on the day of scanning with periodic additional assessments on the remaining days of admission. Some of these patients enrolled in a larger ongoing observational follow-up study.

**Table 2.** Demographic information for patients with DoC

Sex	Age	Months postinjury	Etiology	Diagnosis	CRS-R	Arousal subscore
M	21	45	TBI	MCS+	11	2
M	46	48	TBI	UWS	7	2
M	57	14	TBI	MCS−	12	2
M	55	15	Anoxic	UWS	5	1
M	47	4	TBI	MCS	10	2
M	36	34	TBI	UWS	8	2
M	17	46	Anoxic	UWS	11	2
F	38	13	Anoxic	MCS	11	2
M	29	68	TBI	MCS+	10	2
M	23	4	TBI	MCS	7	2
F	70	11	TBI	MCS	9	2
F	30	6	Cerebral bleed	MCS−	9	2
M	22	5	Anoxic	UWS	7	2
F	62	7	Anoxic	UWS	7	2
M	46	10	Anoxic	UWS	5	2
M	21	7	Anoxic	MCS	11	3
M	67	14	TBI	MCS−	11	2
M	46	23	TBI	UWS	9	2
F	55	6	Hypoxic	UWS	7	2
M	28	14	TBI	MCS	8	2
M	22	12	TBI	MCS+	10	2
F	28	8	Acute disseminated encephalomyelitis	UWS	6	2

Diagnoses were made considering the entire clinical record instead of CRS-R alone. MCS− indicates that patients display visual fixation and pursuit, automatic motor reactions (e.g., scratching, pulling bed sheet), or localization to noxious stimulation. MCS+ classification indicates that patients consistently and repeatedly followed simple commands or intelligibly verbalized (69, 70). Patients classified as MCS showed such behavior but only intermittently. CRS-R is the highest score recorded by the attending physician for the day of the scanning session. CRS-R scores were collected at least once on the day of scanning with periodic additional assessments on remaining visit days.

The subset of *n* = 23 was selected for its suitability for brainstem connectivity analysis in the present study based on strict inclusion criteria: 1) a lack of large focal brain (i.e., more than 1/3 of one hemisphere) and in particular brainstem damage as assessed by a neuroanatomical expert blinded to the patient’s diagnosis, (2) excessive head motion during resting-state scanning (i.e., greater than 3 mm in translation and/or 3° in rotation), and (3) failure of segmentation and normalization during preprocessing. From this selection, one DoC participant was excluded after preprocessing due to unsuccessful image coregistration. A partially overlapping subset has previously been utilized by Luppi et al. (9).

There were no significant differences in age between the healthy control cohort (35 ± 11.448) and DoC patients (39.4 ± 16.5) (*t*(37.506) = −1.022, *P* = 0.313). Among the subset of 22 patients, seven had follow-up rs-fMRI scans with CRS-R assessments (*SI Appendix*, Table S1).

All clinical investigations were conducted in accordance with the Declaration of Helsinki and all relevant ethical guidelines, and a written informed consent of participation was obtained from patients and/or their legal representative/surrogate decision maker. An ethical approval for testing patients was provided by the National Research Ethics Service (National Health Service, United Kingdom; Local Research Ethics Committee reference 99/391). A brief overview of the DoC spectrum can be found in *SI Appendix*.

A subset of the participants who underwent propofol treatment were reported by Adapa and colleagues, who describe acquisition and sedation protocols in detail (58). Briefly, in total, 26 healthy volunteers without a history of neurological disorders (11 male) with a mean age of 34.2 y old (range: 19 to 52 y old) were briefed on the procedures and potential side effects of propofol sedation. Their scans were performed at the Wolfson Brain Imaging Centre, Cambridge, United Kingdom, on a Magnetom Tim Trio 3T (Siemens Healthcare). T1-weighted structural acquisitions were performed using a fast MP-RAGE sequence (TR = 2,250 ms, TE = 2.99 ms, TI = 900 ms, flip angle = 9°, at 1 × 1 × 1 mm^2^ resolution). Functional resting-state was acquired using an echo-planar imaging sequence in four separate sessions consisting of 32 interleaved, descending slices (TR = 2,000 ms, TI = 900 ms, TE = 30 ms, flip angle = 78°, 3 × 3 × 3.75 mm^2^ resolution). Two participants had to be excluded, one due to unsuccessful scan coregistration and the other due to brainstem distortion.

In the sedation experiments, the sessions of resting-state recordings corresponded to dosage levels of the anesthetic agent propofol administered via target-controlled infusion with an Alaris PK infusion pump (Carefusion). The levels recorded were as follows: *no sedation* (Control, 0 µg/mL), *mild* (Ramsey Level 2, 0.6 µg/mL), *moderate* (Ramsey Level 3, 1.2 µg/mL), and a *recovery period* (58). A total of 10 min for the equilibration of plasma levels was allowed after each administration. Then, 5-min rs-fMRI scans were performed. The order of whether moderate or mild dosage was administered first was randomized to control for homeostatic attuning effects.

Two trained anesthesiologists were on site for all recordings, performing propofol administration and Ramsey Alertness/Sedation Scale assessment before and after each scanning run. They monitored all common physiological parameters using an MRI-compatible multiparameter monitor (Precesss, InVivo Corp.), surveilled participants in the scanner, and took two blood samples (2 × 1 mL) in each condition for chromatographic analyses.

Following the resting-state scan, at each sedation level, a semantic judgement task was collected. The experimental procedure is also detailed by ref. 58. Briefly, the task sessions were 5.5 min in duration and made up of alternating 30 s blocks of words and acoustically matched nonspeech buzz or noise stimuli. Stimuli, in blocks of 8 followed by 6 s interim silences, were presented with stimulus onset asynchrony of 3 s in silent intervals between scans. Participants were instructed to identify with a button press whether heard words referred to living or nonliving objects and whether nonspeech auditory stimuli were noise or buzz type. To assess explicit memory formation during each sedation level, subjects were, upon full recovery, presented with familiar (i.e., previously heard; targets/signal) and unfamiliar (not previously heard; distractor/noise) items. Using the signal detection theory model consisting of two normal distributions, one representing signal and one representing noise, we calculated the most commonly used measure of sensitivity, d′, as an approximation of cognitive conscious access (43). d′ is the standardized difference between the means of signal and noise distribution, that is, the higher d′ is, the more readily a signal is detected, as the subject is better able to distinguish old and new items.

Ethical approval for the sedation experiments was obtained from the Cambridgeshire 2 Regional Ethics Committee. Written informed consent for all research was obtained from all participants prior to any experiments.

The TBI patients without disorders of consciousness who received methylphenidate were a subset (*n* = 12, mean age 34.4 ± 12.8) of patients from the larger dataset (*n* = 15), which Manktelow et al. (59) describe in detail. The subselection of *n* = 12 (see Table 3) was based on the same strict inclusion criteria set out for the DoC cohort, with scans from three patients not meeting these. These data were acquired using the same sequences as the DoC patients and their control cohort on two different research visits separated by 2 to 4 wk. TBI patients received 30 mg MPh (30 mg dose visually indistinguishable from lactose placebo) either on their first or second visit, randomized using a Latin square design. Dosages were based on comparable doses used in previous studies in healthy participants as well as National Institute for Health and Care Excellence guidelines for medication in adults (https://www.nice.org.uk). These patients received no other catecholaminergic/dopaminergic agents in the period between research visits. On the visit, 75 min were allowed after MPh administration to ensure that the peak plasma levels of MPh were reached. After these 75 min, the volunteers completed an MRI scan, which included task and resting-state fMRI as well as structural image acquisitions [see Manktelow et al. (59)]. Written informed consent was obtained from all participants and/or legal surrogate decision makers prior to any experiments or brain scans.

**Table 3.** Demographic information for TBI patients who received methylphenidate

Sex	Age	Months postinjury	Etiology	Lesion description	GCS
M	27	25	TBI	Hemorrhagic contusions in bilateral frontal lobes	7
M	53	32	TBI	Right subarachnoid hemorrhage and subdural hematoma	14
M	49	27	TBI	Hemorrhagic contusion left lentiform nucleus	8
F	55	17	TBI	Subarachnoid hemorrhage in left frontoparietal cortex	12
M	29	14	TBI	Hemorrhagic contusions in left temporal lobe/basal ganglia/thalamus	5
M	19	32	TBI	Subarachnoid hemorrhage in left interpeduncular fossa	7
M	21	39	TBI	Multiple petechial hemorrhages, obliterated basal cisterns	3
M	36	11	TBI	Epidural hematoma right temporal lobe	6
M	26	25	TBI	Intraventricular hemorrhage	7
F	34	41	TBI	Intracerebral hemorrhage and right temporal/parietal contusions	NA
M	43	7	TBI	Right subarachnoid hemorrhage and subdural hematoma	10
F	21	9	TBI	Unavailable	NA

The lesion diagnostic description was made by a neurologist and/or neuroradiologist. When NA or unavailable, the injury occurred abroad with detailed records unavailable. GCS = Glasgow coma scale score at time of admission.

### Spatial and Temporal Preprocessing.

Preprocessing for all scans was performed using the CONN functional connectivity toolbox [19c (60)], running in MATLAB (2018b, MathWorks, Inc.).

For spatial preprocessing, functional images were first slice-time corrected, centered to (0,0,0) Montreal Neurological Institute (MNI) coordinates, realigned to correct for movement, and were subjected to Artifact detection tools (ART)-based identification of outlier scans for scrubbing. Following this, the structural image was coregistered to the mean functional image and then segmented and spatially normalized to the MNI-152 template. Functional images were then normalized to the MNI-152 template based on parameters obtained from structural normalization and smoothed with a 6-mm Gaussian kernel at full-width half maximum.

Temporal preprocessing employed masks for white matter (WM) and cerebrospinal fluid (CSF) produced with structural segmentation to regress out physiological noise in the blood oxygenation level dependent (BOLD) signal as it can otherwise influence functional connectivity estimates. This method called CompCor (61) regresses out the first five principal components of WM and CSF signals, movement parameters obtained from realignment, and their first-order derivatives—alleviating the need for global signal regression, which can equally perturb functional connectivity (FC) estimates (62). Additionally, anatomical CompCor scrubs outlier scans that the ART toolbox method has characterized. Thereafter, our data were linearly detrended and filtered using a high-pass filter of 0.008 Hz.

### VTA Primary Functional Connectivity Analyses.

The VTA ROI we used is from the HAAN (39). We chose it based on being a histologically characterized and thus dopamine-specific ROI. Its faithful coregistration to our anatomical and functional scans was extensively assessed visually.

Functional connectivity—the temporal correlation of regional timeseries—is conceptualized to represent information sharing and dynamic cooperation (63), identifying spatially segregated functional units at global scales. Here, FC was calculated using CONN in the form of seed-to-voxel analyses for assessing effects in the whole brain. Temporal correlations for the dopaminergic VTA seed (and in secondary analyses for its “downstream” targets) were computed for all other voxels in the brain using a general linear model (GLM). The functional connectivity analyses produced seed-to-voxel parameter estimate images, which were entered into population-level analyses; we used independent sample Student’s *t* tests to compute differences from healthy controls for DoC patients and paired-sample Student’s *t* tests for differences from the awake state for the propofol dataset. We report results thresholded at voxel level *P* < 0.005 (uncorrected) and cluster level *P* < 0.05 (family-wise error [FWE] corrected for multiple comparisons) for a valid voxel-wise inference approach (64).

### Correlations of VTA Connectivity with “Downstream” Whole-Brain Connectivity of Targets and Behavior.

The clusters of altered VTA connectivity that were revealed in primary functional connectivity analyses were entered as seeds into subsequent seed-to-voxel analyses in their respective cohorts to compute differences from awake controls. This was to reveal altered “downstream” connectivities of VTA targets. All connectivity clusters were extracted as binary masks. To test our hypothesis that VTA connectivity may have neuromodulatory effects on its targets (and resultant network architectures), we extracted eigenvalues of connectivity strength (β-estimates from GLM) per participant per condition for both VTA ⇌ primary-target connectivity and target ⇌ downstream cluster connectivity. To approximate whether there is a neuromodulatory relationship between VTA ⇌ target connectivity alterations and target ⇌ downstream connectivity, we assessed how these two functional connectivity strengths covaried for patients and sedated volunteers, respectively.

All correlations were performed using RStudio with ggplot2 for DoC patients and for propofol experiments with the rmcorr toolbox (65) using analysis of covariance to account for nonindependence among the repeated observations (awake, mild and moderate sedation, and recovery) by statistically adjusting for interindividual variability. For further statistical stringency, the “rmcorr” correlations were bootstrapped (*n* = 1,000) to conform to 95% CIs, and correlations for DoC patients used Spearman’s rank. As behavioral measures, we included the sensitivity index d′ for the propofol sedation dataset and, for patients with DoC, the clinical bedside assessments of highest CRS-R [score and highest CRS-R arousal subscore at time of scan (66, 67)]. Based on CRS-R improvement and clinical assessment, we identified which DoC patients who had follow-up rs-fMRI scans had respectively improved of deteriorated and calculated their VTA–PCu/PCC change (*SI Appendix*, Table SM).

## Supplementary Material

Supplementary File

## Data Availability

The fully processed data and images underpinning the analyses and figures in the present manuscript have been deposited to the repository available at https://doi.org/10.17863/CAM.72170 (68). Due to patient privacy concerns, raw neuroimaging and behavioral data are available upon request by qualified researchers. The UK Health Research Authority mandates that the confidentiality of data is the responsibility of Chief Investigators for the initial studies (in this case, J.A. and D.K.M. and anyone to whom this responsibility is handed—for example, in the context of retirement or transfer to another institution). Requests will be considered on a case-by-case basis, assessing the feasibility and appropriateness of the proposed study, and the capacity to maintain the required levels of data security consistent with the original approved research ethics approval and the patient information sheet that was the basis of consent obtained. The CONN toolbox is freely available online (https://www.nitrc.org/projects/conn). The HAAN atlas, containing the VTA, is freely available from the Athinoula A. Martinos Center for Biomedical Imaging (https://www.nmr.mgh.harvard.edu). The rmcorr and ggplot2 toolboxes are available via CRAN in RStudio (https://www.R-project.org/).
