# Supplementary material for: Dopaminergic brainstem disconnection is common to pharmacological and pathological consciousness perturbation
Source: Proc Natl Acad Sci U S A. 2021 Jul 23;118(30):e2026289118. doi: 10.1073/pnas.2026289118 (PMC8325270; doi:10.1073/pnas.2026289118)
Supplement: Supplementary File [file pnas.2026289118.sapp.pdf]

Dopaminergic brainstem disconnection is common to pharmacological and pathological consciousness perturbation

SI Appendix

Brief description of Disorders of Consciousness

These diseases are classified into strata based on their severity of consciousness disruption. At the most severe end is *coma*, in which both arousal and awareness are low (1). Correspondingly, comatose patients show absence of sleep-wake cycles, have their eyes closed and cannot be aroused by any stimuli, noxious or otherwise.

Instead, those who survive coma most commonly emerge into *unresponsive wakefulness syndrome* (formerly: *vegetative state*) – a pathology in which patients do show sleep-wake cycles and reflexive motor responses to certain stimuli (2). However, these responses are inconsistent and not interactive, indicating that whilst a general level of arousal is preserved, no awareness of self and environment is present (3). Moving up a stratum, patients that do display detectable but atypical signs of fundamental awareness of environment and traces of cognitive functions have been sub-categorized into *minimally conscious state (MCS)*. In clinical assessment, these patients show directed basic motor behaviour, eye-tracking, emotional responses to emotive stimuli, basic vocal communication attempts and binary command understanding although these hallmarks are often difficult to ascertain (4). Correspondingly, baseline arousal is present in MCS patients, paired with a certain (putatively phasically unstable) awareness level (5). In this stratification of DoCs, only one pathology sits above MCS, namely the *locked-in-syndrome (LIS)* in which there is full preservation of awareness and arousal (and therein consciousness) in a however quadriplegic and anarthric body (6). Overall, awareness and arousal are thus not always reliably detectable despite the clear necessity to distinguish different DoCs to inform appropriate care – but equally to assure full consciousness-suppression under anaesthesia. For a comprehensive review of diagnostic criteria, see (6).

Patient	*Diagnosis*	*CRS-R* (1st | 2nd)	*VTA-PCu/PCC FC* (1st)	*VTA-PCu/PCC FC* (2nd)	*Outcome*
1	MCS	(11 | **23**)	-0.0251	**0.0199**	**Alive, improving**
2	MCS	(8 | **11**)	-0.0063	**0.0409**	**Alive, improved**
3	MCS	(10 | 8)	-0.0028	-0.0563	Deceased
4	UWS	(11 | 8)	-0.0025	-0.0197	Deceased
5	UWS	(5 | 4)	-0.0887	-0.0742	Deceased
6	UWS	(7 | 5)	-0.0062	-0.0063	Deceased
7	UWS	(11 | 8)	-0.098	-0.0449	Deceased

Table S1: Diagnoses, clinical scores, outcomes and VTA-PCu/PCC connectivity of patients who had imaging follow-ups. Further demographic or outcome details have been excluded to keep subsample sufficiently anonymised. Improvement in **bold.**

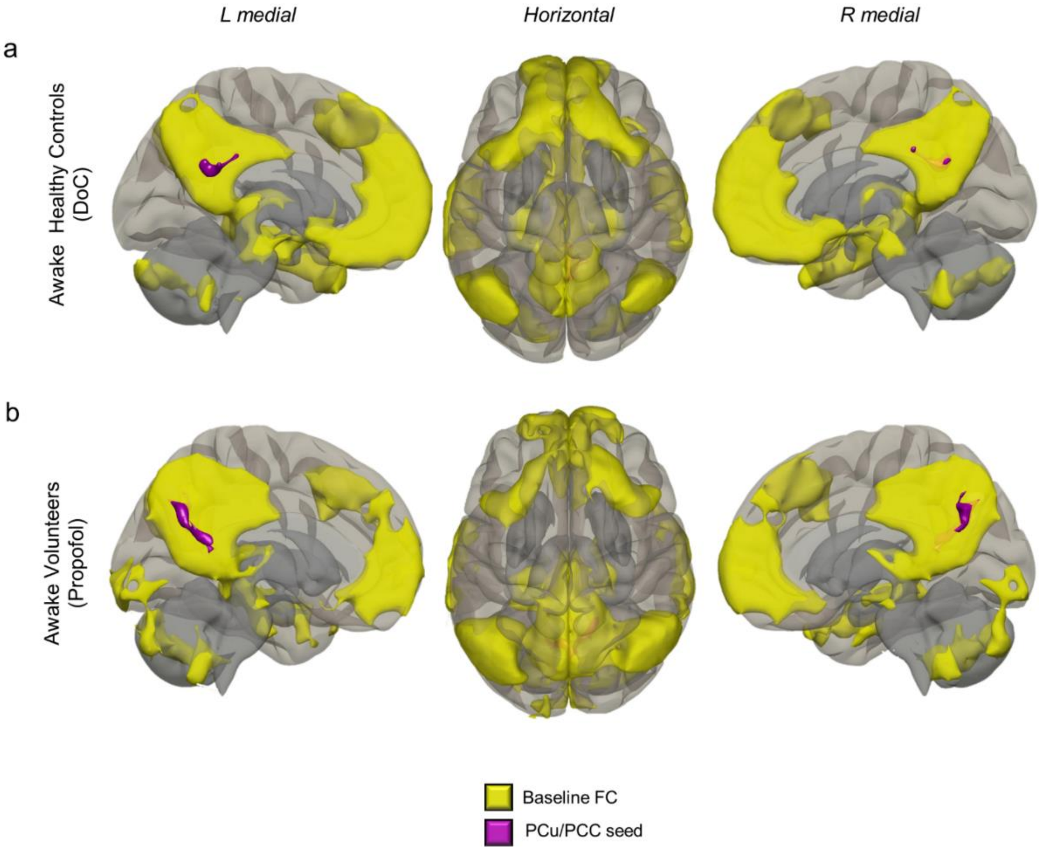

Fig. S1: Resting connectivity of PCu/PCC clusters of VTA disconnection in control cohorts. In new seed-to-voxel analyses in the in (a) the awake controls for the DoC cohort and (b) awake volunteers in the propofol cohort the respective PCu cluster showed baseline connectivity patterns to all canonically identified DMN regions (Buckner & DiNicola, 2019). Connectivities were thresholded at $p<0.001$ voxel-level (uncorrected) and $p<0.05$ cluster-level (FWE-corrected). Renderings were made using the CONN toolbox three-dimension template. L is left.

Table S2: Significant clusters of awake connectivity revealed in seed-to-voxel analyses using the PCu/PCC clusters as seeds in new seed-to-voxel connectivity analyses. Associated CONN, labels, MNI coordinates cluster extent and FWE-corrected p-value are denoted. Results were thresholded at p<0.001 voxel-level (uncorrected) and p<0.005 voxel-level (uncorrected) and p<0.05 cluster level (FWE-corrected). For clusters over 10000 voxels, only labels for regions which occupied over 250 voxels within those clusters are reported.

Condition	*Significant positive FC at rest (CONN-label)*	*Peak MNI Coordinates*	*Cluster size*	*p-FWE corrected*
Awake (Healthy controls DoC dataset)	Precuneus, FP l+r. PC, sLOC l+r, pMTG l, SFG l+r, MidFG l+r, MedFC, AC, PaCiG l+r, SFG l+r, TP l+r, pMTG l+r, SubCalC, AG l+r, Cereb2 l, Hippocampus l+r, Brainstem, Thalamus l+r, aMTG l+r, Cereb9 l+r, pPaHC l+r, Forb l+r, pITG l+r, _aPaHC l+r, Amygdala l+r, Accumbens l+r, LG l+r, Cereb45 l+r, aSTG l+r, IC l+r, pTFusC Cereb8 l+r,	+00 -40 +30	28218	0.000
	pMTG l, TP l, aMTG l, FOrb l, pITG l, aSTG l, aITGl, IC l, toMTG l	-66 -18 -18	3513	0.000
	pMTG r, TP r, aMTG r, FOrb r, pITG r, toMTG r, aSTG r, FP r, aITG r, Amygdala r, pSTG r, IC r	+28 +18 -24	3049	0.000
	sLOC l, AG l	-40 -64 +38	2622	0.000
	sLOC r, AG r	+48 -62 +36	2238	0.000
	Cereb 2 l, Cereb 1 l	-34 -72 -38	989	0.000
	Cereb 2 r, Cereb 1 r, Cereb 7 r, Cereb 8 r	+20 -86 -34	840	0.000
	Cereb 9 l+r, Ver9, Brainstem, Cereb 8 r	-06 -54 -42	762	0.000
	Precuneus, PC, sLOC r, AG r, Cuneal l+r, Hippocampus r, Ver45, LG l+r, pPaHC l+r, Thalamus l+r,	-02 -62 +32	14619	0.000
	ICC l+r, Brainstem, Cereb45 l, AC, PreCG l, SCC l+r, sLOC l, iLOC r, aPaHC l, pTFusC l, SPL r, pSMG r, Ver4, PreCG r	+02 +64 +22	3522	0.000
Awake (Volunteers Propofol dataset)	FP r, MedFC, PaCiG r, FP l, PaCiG l, AC, SubCalC, SFG l+r	-40 -72 +38	3476	0.000
	sLOC l, AG l, SPL l, pSMG l, iLOC l	+22 +38 +42	1681	0.000
	MidFG r, SFG r, FP r	+08 -82 -40	1522	0.000
	Cereb2 l+r, Cereb1 l+r, Cereb7 l+r	-28 +22 +48	1089	0.000
	MidFG l, SFG l, FP l	+66 -30 -10	786	0.000
	pMTG r, aMTG r, pITG r, toMTG r	-60 -04 -22	706	0.000
	pMTG l, aMTG l, TP l	-04 -54 -50	515	0.000
	Cereb9 l+r	+22 -28 -10	275	0.000
	Hippocampus r, pPaHC r, Thalamus r, Brainstem, Amygdala	+06 -96 -12	160	0.002
	OP r, LG r, OP l	+00 +50 +44	120	0.010
	SFG l+r, PaCiG r	+48 +18 -34	115	0.013

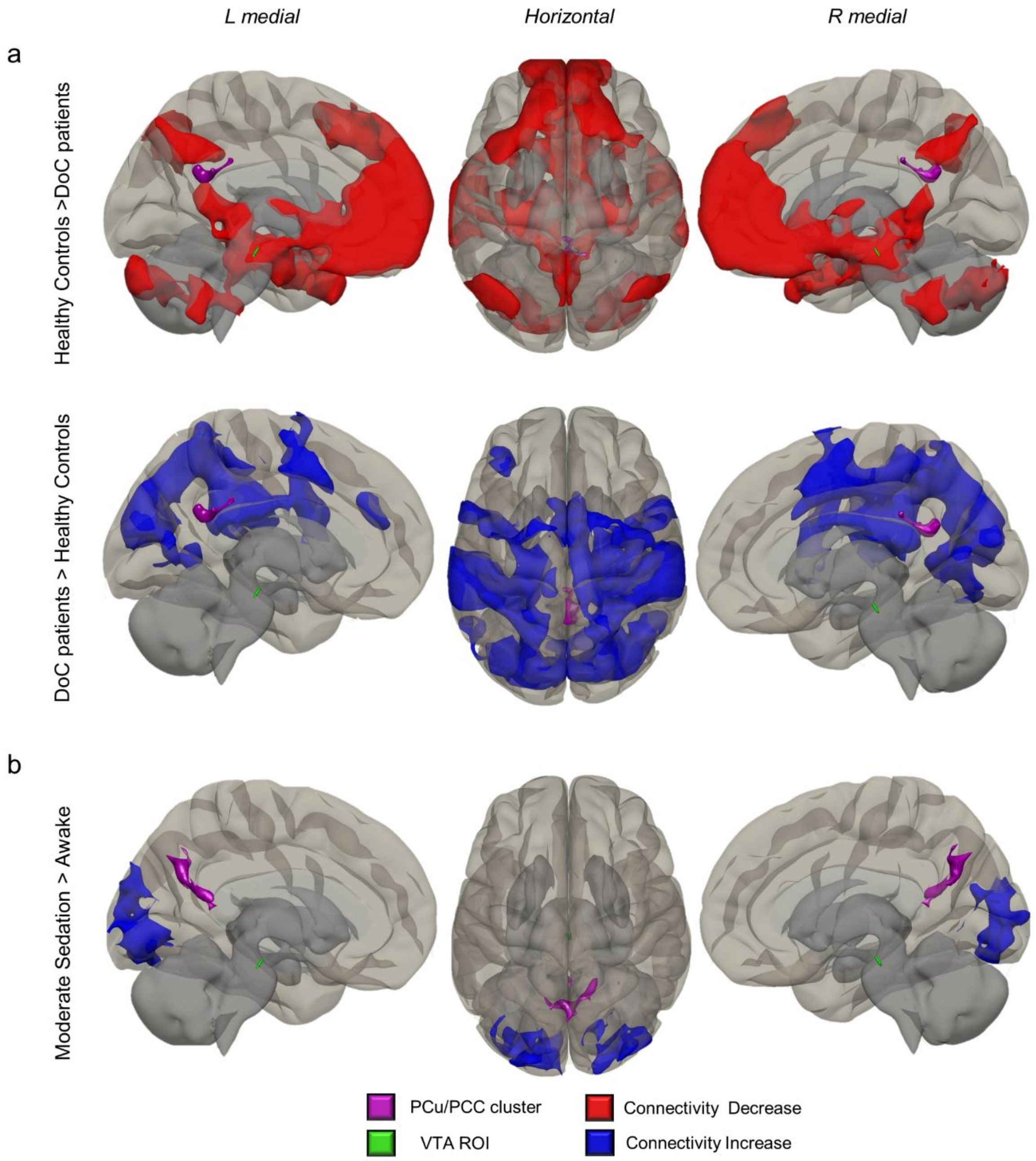

Fig. S2: 'Downstream' connectivity changes of PCu/PCC clusters in pathological and pharmacological states of consciousness perturbation. (a) In a contrast of Healthy controls *versus* DoC patients, the PCu/PCC cluster the VTA had lost connectivity with in DoC patients, showed 'downstream' losses of connectivity (red) with DMN regions, and 'downstream' increases in connectivity with areas not commonly considered part of the DMN (blue). (b) In a contrast of moderate sedation against awake controls, the PCu/PCC cluster the VTA lost connectivity with in Propofol sedation showed significant connectivity increases with an occipital cluster (blue). Renderings were made using the CONN toolbox' 3D template. All connectivity contrasts were thresholded at $p<0.005$ voxel-level (uncorrected) and $p<0.05$ cluster-level (FWE-corrected). L is left.

***Table S3: Significant* clusters of PCu/PCC 'downstream' connectivity changes in contrasts with respective control conditions.** CONN atlas labels, MNI coordinates, cluster extent and FWE-corrected p-values are reported. Results were thresholded at p<0.005 voxel-level (uncorrected) and p<0.05 cluster-level (FWE-corrected). For clusters over 10000 voxels, only labels for regions which occupied over 250 voxels within those clusters are reported.

	**Condition/ contrast**	***ΔFC change***	***Anatomical regions (CONN atlas)***	***Peak MNI Coordinates***	***Cluster size***	***Cluster p value - FWE corrected***
**Disorders of Consciousness**	**Healthy > DoC Patients**	↓Loss	FP l+r, MedFC, MidFG l+r, PC, Precuneus, Brainstem, TP l+r, AC, PaCiG l+r, toMTG l+r, pMTG l+r, Hippocampus l+r, SFG l+r, Thalamus l+r, aPaHC l+r, pPaHC l+r, aMTG l+r, Caudate l+r, Amygdala l+r, FOrb l+r, Accumbens l+r, IC l+r, Putamen l+r, SubCalC, pTFusC, aITG l+r. pITG l+r, aSTG r, LG , Cereb1,3,45,6,8,9,10 l+r, Ver3, 45,8,9 y	-04 +46 -04	32652	0.000
			Cereb1,2,7,8 r	+38 -70 -36	1680	0.000
			Precuneus, PC	+00 -60 +38	987	0.000
			sLOC l, AG l	-44 -72 +48	974	0.000
			sLOC r, AG r	+48 -64 +48	681	0.000
	**DoC Patients > Healthy**	↑Gain	sLOC l+r, PreCG l+r, PostCG l+r, SPL l+r , SMG l+r, SMA l+r , AC, PO l+r, iLOC r, IFG oper r, Cuneal l+r, LG r, PT r, MTG, ICC, OP	-58 -36 +30	28189	0.000
			FP l, MidFG l	-34 +32 +32	390	0.008
			toMTG l, iLOC l	-56 -54 +00	348	0.016
**Propofol Sedation**	**Moderate Sedation > Awake**	↑Gain	OP l, LG l, iLOC l, OFusG, sLOC l, ICC l	-12 -78 -08	1995	0.000
			OP r, iLOC r, OFusG r, sLOC r, ICC r, Cereb 1 r	+40 -80 -06	1192	1192

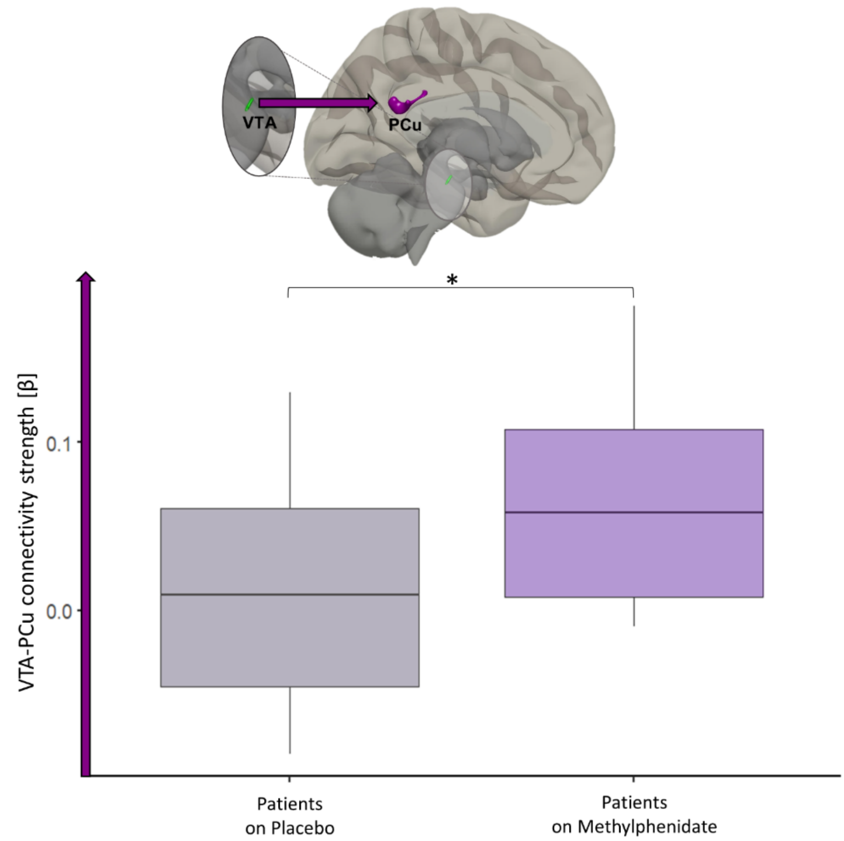

Fig. S3: Boxplot depicting VTA-PCu/PCC connectivity on placebo and on methylphenidate for the 12 TBI patients without disorders of consciousness who received the dopaminergic agonist methylphenidate. Traumatic brain injury/diffuse axonal injury patients (n=12) rs-fMRI scans showed that β-coefficients from the GLM for VTA connectivity to the cluster of PCu/PCC disconnection (purple, purple arrow) observed in DoC patients (Fig.2) was significantly higher ($t(11)=-1.957$, $p=0.038$) in the methylphenidate condition (M=0.065±0.018, magenta) compared to placebo administrations (M=0.011±0.020, grey).

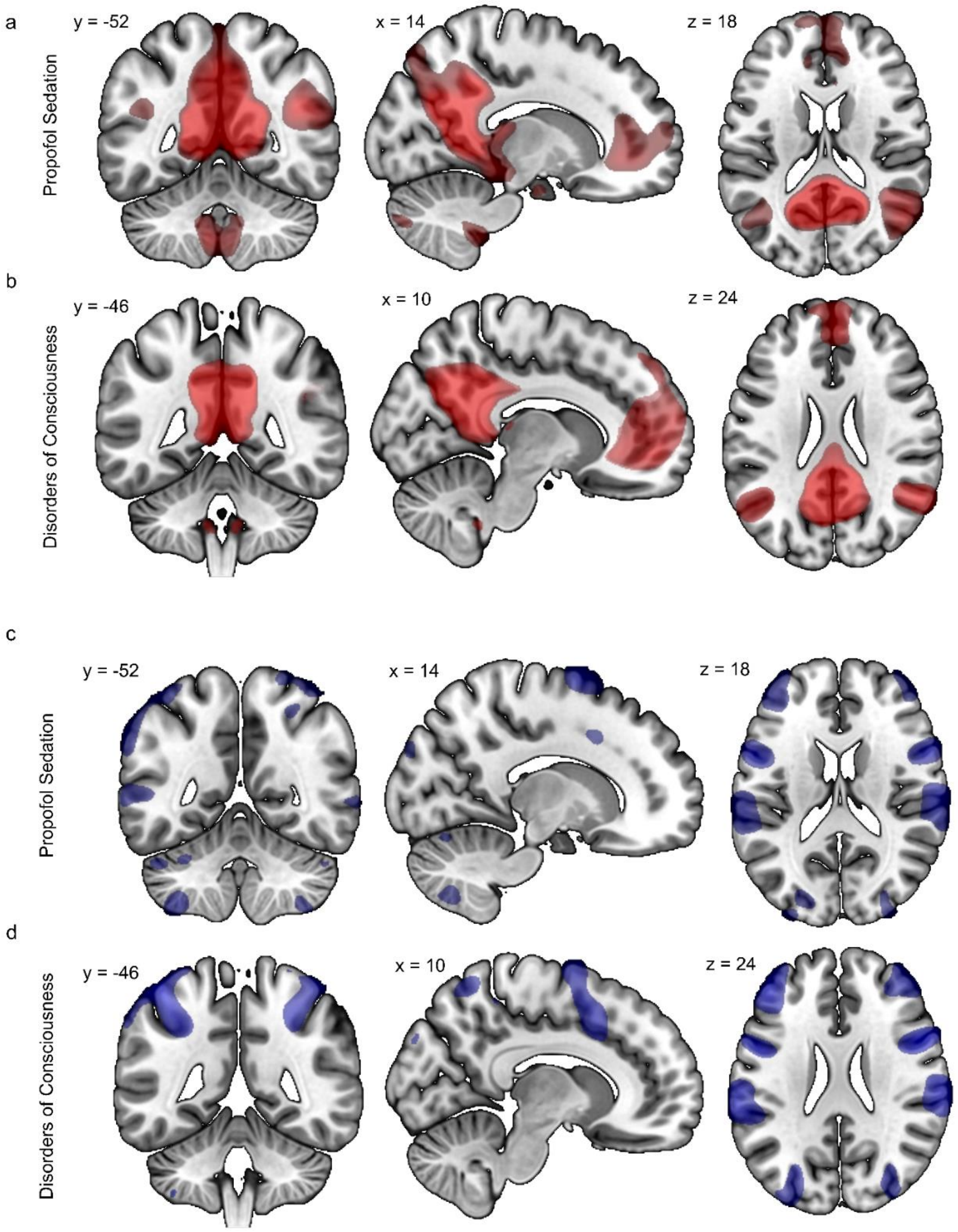

Fig. S4: Neurosynth functional connectivity maps generated with peak MNI coordinates for the respective precuneal/PCC cluster for each cohort. Across both populations, the respective PCu/PCC clusters showed similar positive (a & b) and negative (c & d) functional connectivity patterns. Renderings are centred on peak MNI coordinates. Neurosynth is available as an online tool at: https://neurosynth.org/

References (SI Appendix)

1. S. Laureys, A. M. Owen, N. D. Schiff, Brain function in coma, vegetative state, and related disorders. *Lancet Neurology* **3**, 537–546 (2004).
2. S. Laureys, The neural correlate of (un)awareness: lessons from the vegetative state. *Trends in Cognitive Sciences* **9**, 556–559 (2005).
3. J. T. Giacino, J. J. Fins, S. Laureys, N. D. Schiff, Disorders of consciousness after acquired brain injury: the state of the science. *Nature Reviews Neurology* (2014).
4. L. Naccache, Minimally conscious state or cortically mediated state? *Brain* **141**, 949–960 (2018).
5. K. Khanna, A. Verma, B. Richard, "The locked-in syndrome": Can it be unlocked? *Journal of Clinical Gerontology and Geriatrics* **2**, 96–99 (2011).
6. D. Kondziella, *et al.*, European Academy of Neurology guideline on the diagnosis of coma and other disorders of consciousness. *European Journal of Neurology* **27**, 741–756 (2020).
